# Performance Enhancement of Pedestrian Navigation Systems Based on Low-Cost Foot-Mounted MEMS-IMU/Ultrasonic Sensor

**DOI:** 10.3390/s19020364

**Published:** 2019-01-17

**Authors:** Ming Xia, Chundi Xiu, Dongkai Yang, Li Wang

**Affiliations:** 1 School of Electronic and Information Engineering, Beihang University, Beijing 100191, China; xiaming@buaa.edu.cn (M.X.); edkyang@buaa.edu.cn (D.Y.); 2 Earth Observation System and Data Center, China National Space Administration, Beijing 100101, China; li.gr.wang@gmail.com

**Keywords:** ultrasonic sensor, MEMS-IMU, heading estimation error, process noise covariance, straight motion heading update, fuzzy adaptive extended Kalman filter

## Abstract

The pedestrian navigation system (PNS) based on inertial navigation system-extended Kalman filter-zero velocity update (INS-EKF-ZUPT or IEZ) is widely used in complex environments without external infrastructure owing to its characteristics of autonomy and continuity. IEZ, however, suffers from performance degradation caused by the dynamic change of process noise statistics and heading estimation errors. The main goal of this study is to effectively improve the accuracy and robustness of pedestrian localization based on the integration of the low-cost foot-mounted microelectromechanical system inertial measurement unit (MEMS-IMU) and ultrasonic sensor. The proposed solution has two main components: (1) the fuzzy inference system (FIS) is exploited to generate the adaptive factor for extended Kalman filter (EKF) after addressing the mismatch between statistical sample covariance of innovation and the theoretical one, and the fuzzy adaptive EKF (FAEKF) based on the MEMS-IMU/ultrasonic sensor for pedestrians was proposed. Accordingly, the adaptive factor is applied to correct process noise covariance that accurately reflects previous state estimations. (2) A straight motion heading update (SMHU) algorithm is developed to detect whether a straight walk happens and to revise errors in heading if the ultrasonic sensor detects the distance between the foot and reflection point of the wall. The experimental results show that horizontal positioning error is less than 2% of the total travelled distance (TTD) in different environments, which is the same order of positioning error compared with other works using high-end MEMS-IMU. It is concluded that the proposed approach can achieve high performance for PNS in terms of accuracy and robustness.

## 1. Introduction

Location-based services (LBS) are closely related to industrial production and daily life in an intelligent and information society. A precise location is the basis for providing a high-quality LBS. In outdoor scenes, global navigation satellite system (GNSS), which includes GPS, Beidou, GLONASS and Galileo, has been used widely and successfully [1]. However, satellite signals are easily blocked and affected by the multipaths inside buildings where humans spend most of their time. Therefore, the techniques for indoor environments have flourished, two of which are infrastructure-based and infrastructure-free indoor positioning systems. Infrastructure-based methods, such as Ultra-Wide Band (UWB) [2,3], WIFI [4,5], RFID [6], Zigbee [7], magnetic field [8,9], etc., can provide sufficient positioning accuracy for common applications; however, network devices must be pre-installed in buildings, and unfortunately will be unavailable in complex environments without GNSS signals, such as fire scenes and underground locations. Infrastructure-free methodology, such as microelectromechanical system inertial measurement units (MEMS-IMUs), in contrast, require no external facility support. Due to advancements in microelectronics and micromachining technologies, inertial sensors have evolved, featured by compactness, low power and affordable grade, and have become popular in civil and consumer markets.

A MEMS-IMU is comprised of three gyroscopes and three accelerometers, and can provide pedestrians with autonomous solutions in different types of environments; nevertheless, as a kind of relative localization method, it is confronted with the problem of error accumulation. For example, due to the absence of references to assist MEMS-IMU, the positioning error can be more than 100 m in ten seconds [10]. Usually, human trajectories are estimated by the IEZ framework which includes an inertial navigation system (INS), extended Kalman filter (EKF) and zero velocity update (ZUPT) [11,12]. The measurement update in EKF takes pseudo-measurements of velocity by ZUPT every time the foot moves with respect to the ground. Position errors over time increase from cubic to linear. In [13], the information extracted from an additional chest-attached accelerometer is used to determine the threshold for the still-phase detection. ZUPT can supply effective measurements for EKF even if the walking velocity changes rapidly. In Ref. [14], a model of the acceleration bias error estimate is developed during the swing phase when ZUPT does not work. Velocity and position can be corrected in the whole gait cycle. In Ref. [15], a two-stage EKF is designed for the integration of gyroscopes, an accelerometer and a magnetometer. The proposed filter precludes the impacts of magnetic anomalies. In Ref. [16], in the case of long-distance tracking and different walking gait patterns, a high-end MEMS-IMU achieves ideal positioning performance which errors as a percentage of the total travelled distance (TTD) range from 0.32% to 1.04% in the experiment. Nevertheless, the devices are as expensive as the method in Ref. [16]. In Refs. [11,12,13,14,15], only process noise and measurement noise follow a Gaussian distribution, and the corresponding statistics are constant and accurately known so that optimal estimation can be realized for the pedestrian navigation system (PNS). However, in practical applications, obvious discrepancy between real states and estimates always exists because the prior assumptions of the process noise covariance matrix *Q* hardly remain unchanged. This change is caused by the fact that the statistic noise levels easily suffer from the nature and the quality of low-cost MEMS-IMU. Hence, the performance of the conventional EKF is deteriorated as a result of fixed *Q*.

To address the problem of unfaithful process noise statistics and improve the robustness of EKF, the H-infinity filter with a cost function of minimizing the maximum estimation error is used to provide a robust state estimation concerning the noise uncertainty in the system, though additional analysis or computation is required [17,18]. Furthermore, many adaptive methods are applied for the location accuracy and robustness. In Ref. [19], an adaptive fading memory filter is proposed to form a scale factor to deliberately revise the state prediction covariance and reduce the influence of historical observation data. In Ref. [20], output residuals show similar properties to Gaussian white noise through orthogonality, due to a strong tracking filter (STF). For the whole recursive filtering process, the suboptimal fading factor, which may lead to the loss of accuracy when unknown situation exists, is involved [19,20]. In Ref. [21], the principle of multiple model adaptive estimation (MMAE) runs a bank of Kalman filters with different stochastic models in parallel and selects the best one, but this principle is suitable only for the case that one of the models in all running filters is correct. In Ref. [22], an innovation adaptive estimation (IAE) constructs an innovation variance sequence, measuring the changes of innovation to correct the EKF gain matrix directly. In the past decades, many researchers hold that fuzzy inference system (FIS) is close to human thinking and therefore FIS has become a powerful tool to overcome EKF limitations [23,24,25]. Compared with the traditional adaptive EKF method, the main advantages of FIS lie in its simplicity without the need for a precise model. In Refs. [26,27], FIS is exerted on autonomously tuning noise statistical parameters to increase the accuracy and the robustness of the EKF based on IAE. Though these filtering algorithms with robustness have been successfully utilized in different scenarios, they are seldom considered for PNS in harsh environments. In a sense, this is highly inspired by the works in Refs. [22,23,24,25,26,27]. Therefore, we propose a fuzzy adaptive extended Kalman filter (FAEKF) method for PNS to address the uncertain process noise. FAEKF indicates that noise parameters are updated according to the scaling factor of FIS feedback. For the uncertain process noise covariance matrix *Q* in PNS, FIS is exploited to adjust *Q* to prevent the divergence of EKF and ensure the robustness of positioning.

The heading direction finds the accumulation of large errors, and is one of the main sources of errors for PNS because the gyroscopic bias is not observable from ZUPT measurements alone. Refs. [28,29] adopt a heuristic drift reduction (HDR) method with straight-line features of many indoor walkways. Once the information that the user is walking straight is detected, HDR will return an amendment value to reduce the drift of gyro output. The drawback is that this algorithm cannot make a distinction between the drift and actual curving motion. In Ref. [30], the magnetic field sensed by the magnetometer can be used as a reference measurement for the absolute heading, but the magnetometer fails to work well under magnetic disturbance. In Ref. [31], a LiDAR sensor is integrated with MEMS-IMU to calculate the direction of nearby walls that provide heading corrections. However, LiDAR sensors are costly and bulky for PNS applications. In Ref. [32], firefighters deployed ultrasound beacons and a sensors-network is constituted to provide bearing measurements that will help in minimising the drift of inertial estimates. Unfortunately, the deployment of beacons is an added task to the firefighters. To solve the problem of heading drift, straight motion heading update (SMHU) is developed in this study. SMHU improves heading accuracy by an additional ultrasound sensor that detects whether a straight walk happens.

**Figure 1:**
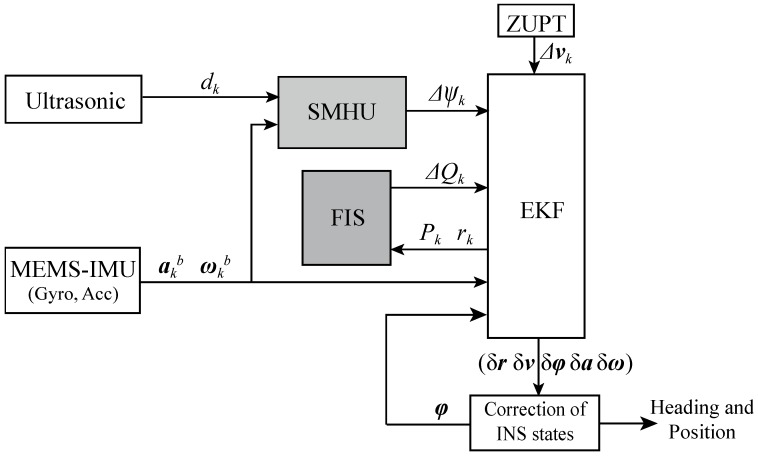
Structure of proposed positioning system.

The contributions of this study as follows: (1) the robustness of PNS is improved by tuning the process noise covariance matrix *Q* of EKF through fuzzy logic techniques; (2) the heading estimation error is reduced with SMHU based on the integration of the low-cost foot-mounted MEMS-IMU and ultrasonic sensor. In order to achieve high performance for PNS in terms of accuracy and robustness, we integrate MEMS-IMU and ultrasonic sensor for PNS as shown in Figure 1.

This system includes two kinds of sensors: MEMS-IMU and ultrasound sensors. MEMS-IMU provides 3-axis accelerometer, gyroscope and magnetometer readings which are (*f*_x_, *f*_y_, *f*_z_), (*w*_x_, *w*_y_, *w*_z_) and (*mag*_x_, *mag*_y_, *mag*_z_) in the coordinate system of the sensor, respectively. The ultrasound sensor calculates the distance between the wall and pedestrian.

The basic structure of a foot-mounted positioning system is IEZ. The state information calculated by a strapdown solution is corrected by the EKF. The EKF is assisted by FIS for covariance matrices of process noise changed sometimes dynamically during filter operation. The ultrasound sensor is also used to correct heading because errors in the yaw orientation are not observable from ZUPT measurements alone.

The rest of this paper is organized as follows: in Section 2, we describe the INS mechanization, error model of MEMS-IMU and EKF. In Section 3, the method for detecting linear features through the ultrasonic sensor is given. In Section 4, FIS is used to correct of process noise covariance matrices. The experimental result and numerical study are reported in Section 5. Conclusions are given in Section 6.

## 2. Theoretical Background of PNS

**Figure 2:**
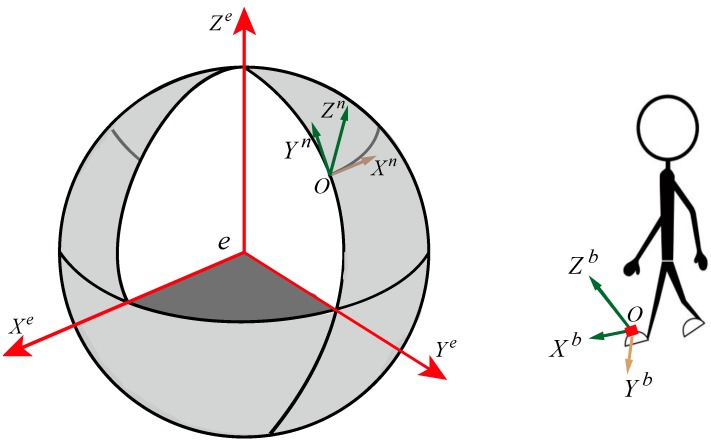
The reference frames involved in the algorithm.

The coordinate system plays an important role in the inertial navigation algorithm of PNS. Two main coordinate frames are employed in this study. In Figure 2, the body frame {b} is formed by the axes of MEMS-IMU, fixed on the right foot of the pedestrian. The navigation frame or ENU frame {n} is a locally defined frame whose axes are consistent with the east, north, and up directions, respectively. The output of the sensor at every sample interval from {b} system is transformed into {n} system to solve navigation parameters. The body frame and navigation frame are identified with the superscripts b and n, respectively.

### 2.1. Basic MEMS-IMU Mechanization

MEMS-IMU mechanization equations express the navigation states of PVA (position, velocity and attitude). Navigation parameters are solved in the PNS algorithm using accelerometer and gyroscope readings from MEMS-IMU.

The differential equation of attitude is:

(1) $${\dot{C}}_{b}^{n}=C_{b}^{n}\Omega^{b}$$

where Ω*^b^* is the skew symmetric matrix of angular rates:

(2) $$\Omega^{b}=\left[\begin{array}{ccc} 0 & -\omega_{z} & \omega_{y}\\ \omega_{z} & 0 & -\omega_{x}\\ -\omega_{y} & \omega_{x} & 0 \end{array}\right]$$

In Equation 1, $C_{b}^{n}$ is the transformation matrix from {b} frame to {n} frame; *w_x_*, *w_y_* and *w_z_* are gyroscopic measurements from *x*-axis, *y*-axis and *z*-axis in {b} frame, respectively.

The differential equation of velocity is:

(3) $${\dot{V}}^{n}=C_{b}^{n}f^{b}-\left(2\omega_{ie}^{n}+\omega_{en}^{n}\right)\times V^{n}+g^{n}$$

where *V^n^* is the velocity vector (*v_x_*, *v_y_*, *v_z_*), and *f^b^* is the specific force vector (*f_x_*, *f_y_*, *f_z_*) measured by the accelerometers. Compared with traditional inertial algorithms, some simplifications are applied for the PNS algorithm based on the characteristics of MEMS-IMU. MEMS-IMU is insensitive to the earth rate $w_{ie}^{n}$ and the rate of {n} frame with respect to {e} frame. $w_{en}^{n}$ is not considered in PNS, so the part (2$w_{ie}^{n}$ + $w_{en}^{n}$) × *V^n^* is omitted, and the gravity *g^n^* can be regarded as a constant vector.

The position can be updated by applying the difference equation:

(4) $${\dot{R}}^{n}=V^{n}$$

where *R^n^* is the position vector (*x*, *y*, *z*).

### 2.2. Error Model of MEMS-IMU

The positioning accuracy of low-cost MEMS-IMU is mainly limited by cumulative errors. It is generally more interesting from the perspective of positioning to consider the time evolution of the corresponding errors. The differential equation of errors, the model in which errors change with time, can be obtained through the first order approximation, ignoring the higher order small quantity introduced in Section 2.1. We include a slightly simplified form as follows:

(5) $$\delta {\dot{R}}^{n}=\delta V^{n}$$

(6) $$\delta {\dot{V}}^{n}=C_{b}^{n}\delta f^{b}-C_{b}^{n}{\left[f^{b}\right]}_{\times }\delta \varphi =C_{b}^{n}\delta f^{b}+\delta C_{b}^{n}f^{b}$$

(7) $$\delta {\dot{\psi }}^{n}=C_{b}^{n}\delta \omega^{b}$$

where *δR^n^* is the position error state; *δV^n^* is the velocity error state; *δψ^n^* is the attitude error state; *δw^b^* and *δf ^b^* are gyro drift and accelerometers, respectively.

Although the above error equations are important, the key point is that the performance of PNS is ultimately affected by errors from accelerometer and gyro. Measurement errors consist of deterministic and random errors. The former can be calibrated by special devices and then removed from raw measurements, while the latter should be modeled stochastically to mitigate their deleterious effect on positioning accuracy. A random walk (RW) process is applied for low-cost MEMS-IMU [23,24]. The RW is represented by:

(8) $$\{\begin{array}{c} \delta {\dot{\omega }}^{b}=w_{g}\\ \delta {\dot{f}}^{b}=w_{a} \end{array}$$

where both *w_g_* and *w_a_* are zero-mean Gaussian white noise.

### 2.3. EKF

The basic concept of EKF is to perform a linear approximation to a nonlinear function, neglecting the higher order terms. This kind of filtering method is effective for situations with a nonlinear system model. Linearization is required for the case of the mechanization equations of MEMS-IMU, as shown in Section 2.1.

EKF relies on a system model and a measurement model. The system model describes the state based on the value of the previous time to update with kinematics or other principles; additionally, the measurement model refers the relationship between one-step estimation state and measurements. In the proposed algorithm, the 15-element error state vector is defined as follows:

(9) $$\delta X_{k}={\left[\begin{array}{ccccc} {\left(\delta R_{k}^{n}\right)}^{T} & {\left(\delta V_{k}^{n}\right)}^{T} & {\left(\delta \psi_{k}^{n}\right)}^{T} & {\left(\delta f_{k}^{b}\right)}^{T} & {\left(\delta \omega_{k}^{b}\right)}^{T} \end{array}\right]}^{T}$$

Having defined the error state vector at time *k*, the linearized system model becomes

(10) $$\delta X_{k+1}=\Phi_{k}\delta X_{k}+G_{k}w_{k}$$

where Φ*_k_* denotes the 15 × 15 state error transition matrix, *w_k_* is Gaussian white noise with zero mean, and the process noise covariance matrix is defined as:

(11) $$E\left(w_{k}w_{j}^{T}\right)=\{\begin{array}{c} Q_{k},k=j\\ 0,others \end{array}$$

(12) $$\Phi_{k}=\left[\begin{array}{ccccc} I & T_{s}\cdot C_{b_{k,k-1}}^{n} & O & O & O\\ O & I & -T_{s}\cdot C_{b_{k,k-1}}^{n}{\left[f^{b}\right]}_{\times } & T_{s}\cdot C_{b_{k,k-1}}^{n} & O\\ O & O & I & O & T_{s}\cdot C_{b_{k,k-1}}^{n}\\ O & O & O & I & O\\ O & O & O & O & I \end{array}\right]$$

where *T_s_* is time interval; *I* is 3 × 3 order zero matrix; *O* is 3 × 3 order unit matrix; [*f*^b^]_×_ is the skew symmetric matrix:

(13) $${\left[f^{b}\right]}_{\times }=\left(\begin{array}{ccc} 0 & -f_{z_{k}}^{b} & f_{y_{k}}^{b}\\ f_{z_{k}}^{b} & 0 & -f_{x_{k}}^{b}\\ -f_{y_{k}}^{b} & f_{x_{k}}^{b} & 0 \end{array}\right)$$

The EKF measurement model is given by:

(14) $$Z_{k}=H_{k}\delta X_{k}+v_{k}$$

where *H_k_* is the measurement matrix; *v_k_* is Gaussian white noise with zero mean, unrelated with *w_k_* at any time; the covariance matrix of measurement noise is expressed as:

(15) $$E\left(v_{k}v_{j}^{T}\right)=\{\begin{array}{c} R_{k},k=j\\ 0,others \end{array}$$

Equations of EKF are divided into two groups: time update and measurement update [25]. The time update equations are one-step prediction with respect to state and covariance:

(16) $$\delta {\hat{X}}_{k}^{-}=\Phi_{k-1}\delta {\hat{X}}_{k-1}^{+}$$

(17) $$P_{k}^{-}=\Phi_{k}P_{k-1}^{+}\Phi_{k}^{T}+G_{k}Q_{k}G_{k}^{T}$$

where (−) is the estimated value after one-step prediction; (+) is the estimated value after the measurements adopted during update; *P* is the estimated covariance matrix of PNS; *G* is the driven matrix.

The measurement update equations utilize new measurements with the time updating results to obtain the optimized posteriori state and covariance. The following are given:

(18) $$K_{k}=P_{k}^{-}H_{k}^{T}{\left(H_{k}P_{k}^{-}H_{k}^{T}+R_{k}\right)}^{-1}$$

(19) $$\delta {\hat{X}}_{k}^{+}=\delta {\hat{X}}_{k}^{-}+K_{k}\left(m_{k}-H_{k}\delta {\hat{X}}_{k}^{-}\right)$$

(20) $$P_{k}^{+}=\left(I-K_{k}H_{k}^{T}\right)P_{k}^{-}$$

where *m_k_* is the actual error measurement; *K* is the Kalman gain matrix that weighs the relative importance of predictions from the dynamic model of the system against pseudo measurements. Pseudo measurements indicate that the measured information is not provided by a meter but by the mathematical calculation and heuristic method.

The actual error measurement *m_k_* at time *k*, which plays the role of feeding EKF, is calculated by ZUPT, zero angular rate update (ZARU) [12] and SMHU. If the foot mounted with MEMS-IMU and ultrasonic sensor touches the ground, the velocity, angular rates and the difference of the angular rate between steps are almost zero:

(21) $$m_{k}=-{\left[\begin{array}{ccc} v_{k}^{T} & \omega_{k}^{T} & \Delta \psi_{k} \end{array}\right]}^{T}$$

We employ ZUPT, ZARU and SMHU methods for pseudo measurements. The measurement matrix *H_k_* is 7 by 15 matrix:

(22) $$H_{k}=\left[\begin{array}{ccccc} O_{3\times 3} & I_{3\times 3} & O_{3\times 3} & O_{3\times 3} & O_{3\times 3}\\ O_{3\times 3} & O_{3\times 3} & O_{3\times 3} & O_{3\times 3} & I_{3\times 3}\\ O_{1\times 3} & O_{1\times 3} & \left[001\right] & O_{1\times 3} & O_{1\times 3} \end{array}\right]$$

## 3. Straight Motion Heading Update (SMHU)

**Figure 3:**
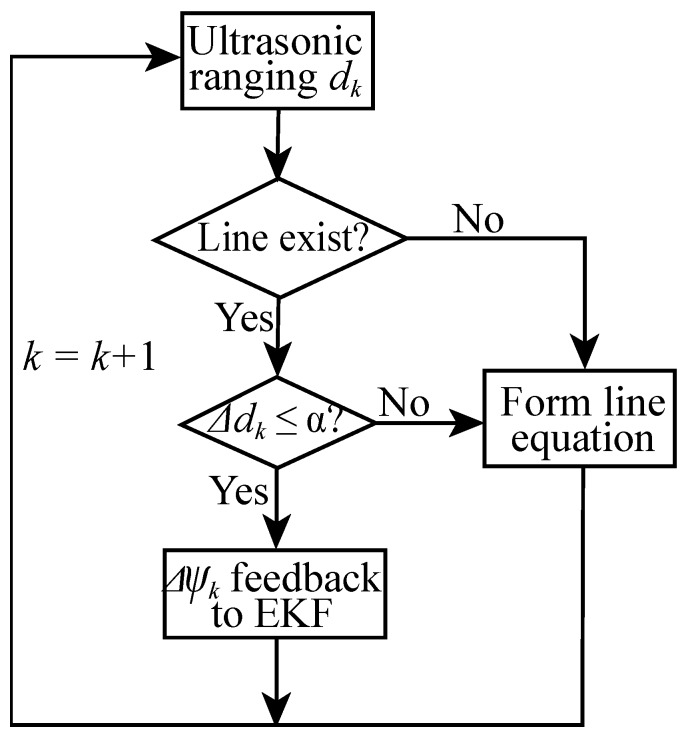
Flowchart of SMHU at time *k.*

The heading error and the heading gyro bias are the only important states which are not observable by applying ZUPT [8,12,33]. For most buildings and walls constructed in straight lines, humans habitually walk along the walls and thus their trajectories are always straight except their walks into corners. When pedestrians present this type of line tracks on corridors or hall ways, this situation can be detected and exploited to reduce the errors in a gyro heading angle. Therefore, we propose an improved method “SMHU” that detects whether a straight walk happens by analyzing the distance between the ultrasonic sensor mounted on the foot and wall. If the detected distances during the first two steps satisfy some conditions, a straight line can be coupled. Once the position of the next step is located on this line, SMHU will be employed and the linear features will feed EKF to mitigate the heading errors. Figure 3 shows the flowchart of SMHU at time *k* to assist EKF.

**Figure 4:**
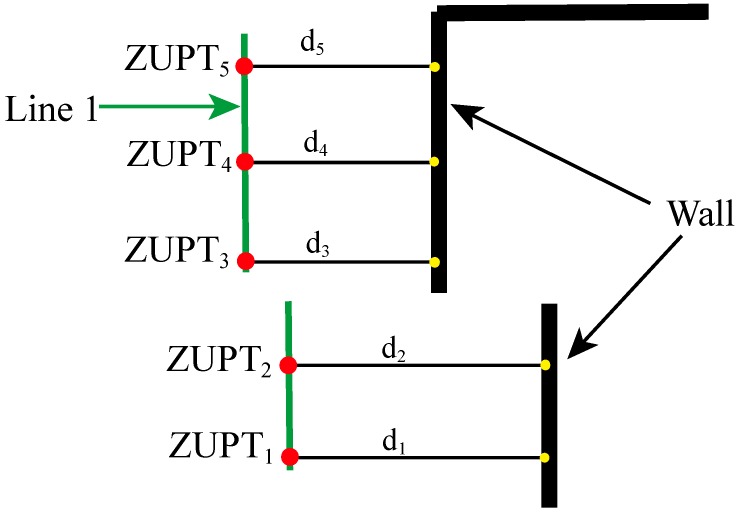
Straight line detection and formation by ultrasonic ranging and MEMS-IMU.

Linear feature extraction for walking in the indoor environment is explained in Figure 4 with an example. Red dots represent the feet on the ground during the walking stance. Black thick lines and yellow points represent the walls on the corridor and reflection of ultrasonic ranging, respectively. During the tests, the right foot equipped with sensors touches the floor almost periodically. ZUPT*_i_* stands for the foot falling to the ground at the *i*th time, and the values for velocity and angular rate are zero during this period. *d_i_* represents the distance measured by the ultrasonic sensor between the foot and reflection point of the wall. Suppose that a person stays at ZUPT_5_, and then *d*_5_ is obtained from the ultrasonic sensor. If *d*_5_ is close to *d*_3_ and *d*_4_, the position of ZUPT_5_ matches the equation of line 1 formed by the previous two points (ZUPT_3_ and ZUPT_4_). Then we can feed this information to EKF to correct the heading error. If *d*_5_ is far away from *d*_3_ and *d*_4_, the linear equation should be refined by ZUPT_5_ and ZUPT_6_ to determine whether the third point ZUPT_7_ belongs to this line.

**Figure 5:**
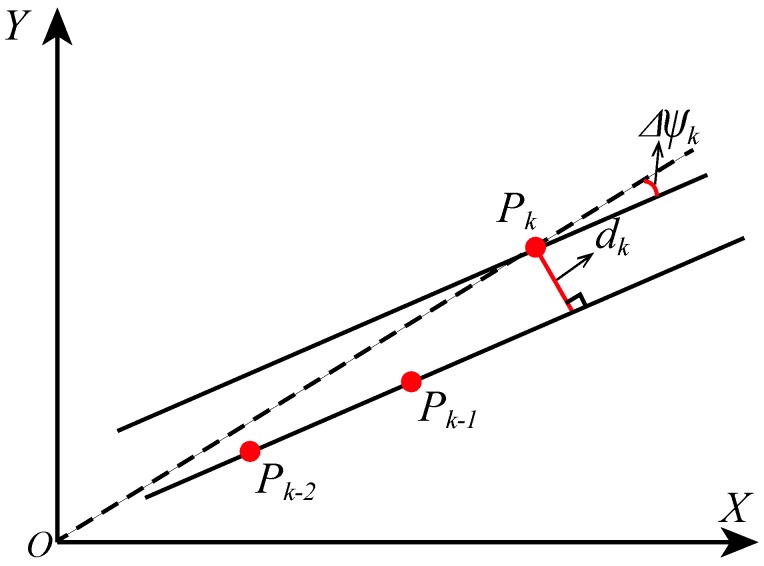
Calculation of heading drift due to gyro bias.

Figure 5 shows the distance from the linear equation: *y* + *Ax* + *B* = 0 to point *P_k_*. The *X* axis and the *Y* axis represent the eastward and northward directions of the navigation coordinate system, respectively. Δ*ψ_k_* is the angle change of the current position relative to the straight line. Assume that the current time is *k*, and then we calculate the location of the foot *P_k_* based on IEZ. The linear equation parameters (*A*, *B*) are computed from the positions of previous two steps, *P_k_*_−1_ and *P_k_*_−2_.

If the distance between the line and point, Δ*d_k_*, is small enough (below a given threshold *α*), there is a straight-line walking mode. Whether the point *P_k_* belongs to the line is determined by the following inequality:

(23) $$\Delta d_{k}=\frac{\left|y_{k}+Ax_{k}+B\right|}{\sqrt{1+A^{2}}}\leq \alpha$$

If *P_k_* belongs to the line, the measurement Δ*ψ_k_* provides feedback for EKF to revise the heading error and make the trajectory straight:

(24) $$\Delta \psi_{k}=\psi_{k}-\tan^{-1}\left(-A\right)$$

If Δ*d*_k_ is larger than the threshold *α*, then the orientation change is considered to be a real variation in the trajectory of a person. In that case, no corrections about the heading error are sent to EKF.

**Figure 6:**
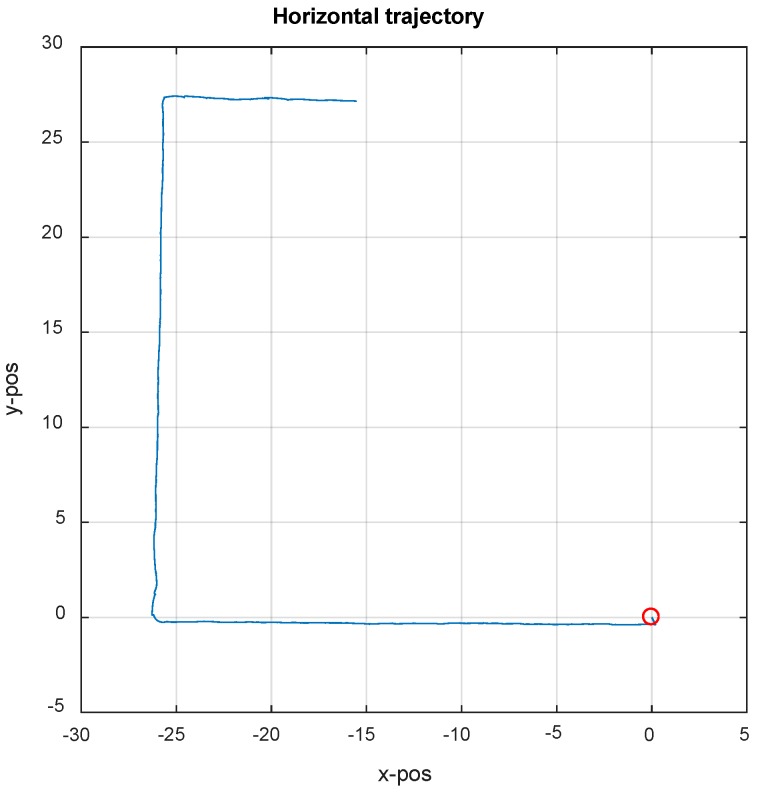
Horizontal trajectory based on IEZ with SMHU.

**Figure 7:**
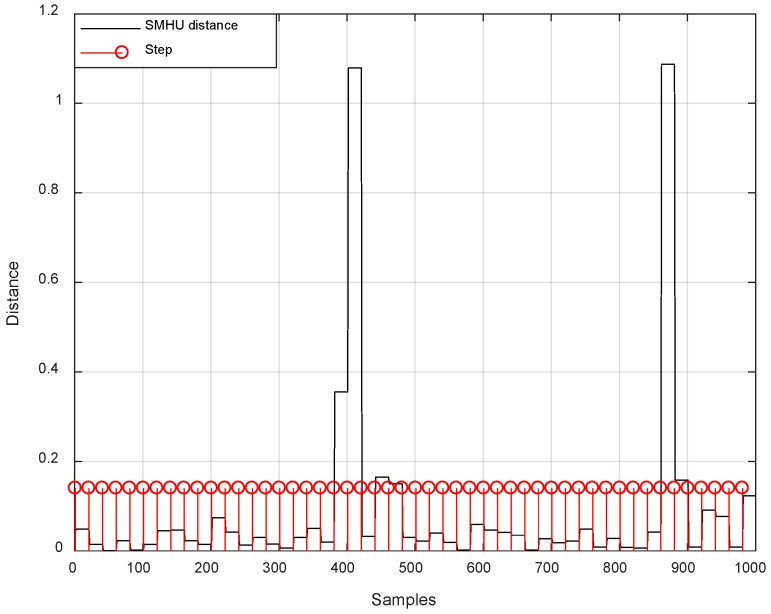
Corresponding distance change at each foot step by ultrasonic sensor.

Figure 6 shows the experiment carried out around the corridor on the fourth floor of New Building at Beihang University. At the beginning, we start from the origin (red circle), walk along the straight corridor, and then alternately turn and walk straight. The SMHU algorithm detects the distance change between the current step and the straight line coupled with the previous two steps (with a threshold of 15 cm). If the difference in detection distance exceeds the threshold in Figure 7, the drift from the estimated trajectory will be reduced by applying SMHU continuously in the proposed algorithm.

## 4. The Adaptive Tuning EKF Algorithm

**Figure 8:**
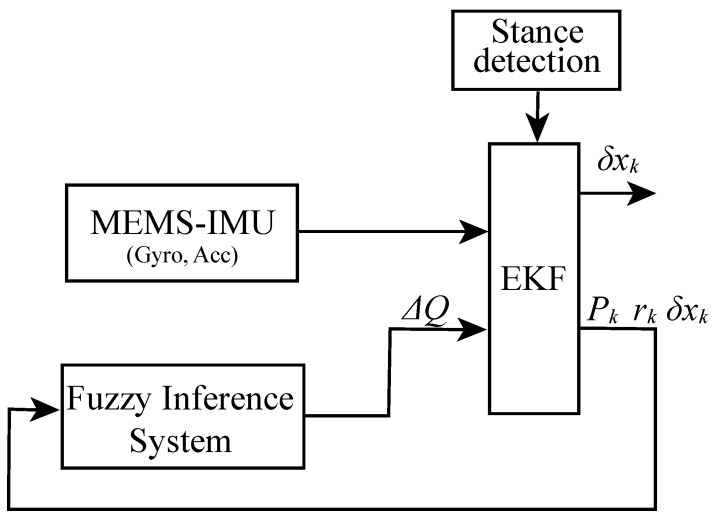
Block diagram of fuzzy adaptive EKF (FAEKF) for PNS.

The optimality of EKF depends on the assumption that system equations and priori noise properties are known and remain constant. However, these conditions are hardly maintained in practice due to the state of motion, running time and environmental factors, etc. One of limitations is the dynamic change of the process noise covariance matrix *Q* as the moving distance increases because of the nature of the low-cost MEMS-IMU. *Q* provokes system estimation state deviation and influences the weight that the filter applies the one-step process information. Errors from this parameter may result in suboptimal EKF, or even seriously deteriorated performance. To improve the robustness of EKF, most of the work reported in this area has concentrated on IAE [25]. The scaling factor is constructed directly from the innovation to correct *Q* adaptively. Some scholars [22] have also compensated the measurement noise covariance matrix with FIS and innovation to cope with the change of measurement noise. This study proposes FAEKF, the IAE approach coupled with fuzzy logic techniques, to adjust *Q* of EKF in the PNS system. Figure 8 shows the block diagram of FAEKF based on PNS.

**Figure 9:**
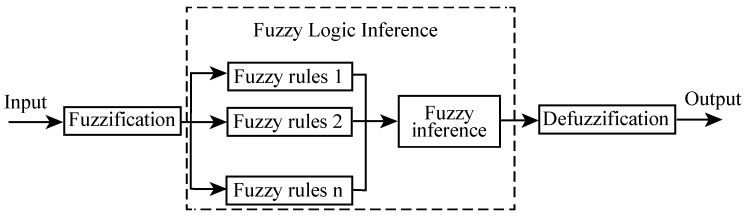
Structure of fuzzy inference system (FIS).

FAEKF can improve the robustness of EKF without loss of accuracy, as FIS can be used to identify the dynamic or incorrect variation of *Q*. FIS is a rule-based expert method that can mimic human thinking and understand linguistic concepts [27]. FIS architecture mainly includes three parts: fuzzification, fuzzy logic inference, and defuzzification as shown in Figure 9.

### 4.1. Fuzzification

Fuzzification is the procedure mapping the numerical variables of input into fuzzy variables. Each linguistic variable corresponds to a series of fuzzy subsets and its MFs. Membership function, which ranges from zero to one, represents the membership grade of any element in the universe of discourse that belongs to a fuzzy set. This function is also the core of a fuzzy set. Values of fuzzy subsets are words or terms from natural or artificial languages, such as “large”, “medium” or “small”.

The input of FIS is the rough ratio between statistical sample covariance of innovation and its theoretical covariance at each step. Assuming that the measurement noise covariance matrix *R* is known and constant, the innovation *r_k_* represents the difference of state estimation error and observation error at time *k*. Statistical sample covariance of innovation ${\hat{C}}_{k}$ is calculated by a limited number of innovation samples. Theoretical covariance *C_k_* is related to the process noise covariance matrix *Q* and the measurement noise covariance matrix *R*:

(25) $$r_{k}=Z_{k}-H_{k}\delta X_{k}^{-}$$

(26) $${\hat{C}}_{k}=\frac{1}{N}\sum_{i=k-N+1}^{k}\left(r_{i}^{T}r_{i}\right)$$

(27) $$C_{k}=H_{k}\left(\Phi_{k}P_{k-1}^{+}\Phi_{k}^{T}+G_{k}Q_{k}G_{k}^{T}\right)H_{k}^{T}+R_{k}$$

where *N* is the size of the moving window. Adopting FIS for FAEKF in this study is to match statistical sample covariance of innovation ${\hat{C}}_{k}$ with its theoretical value *C_k_*. Therefore, we define the input of FIS, *Idm_k_* as follows:

(28) $$Idm_{k}=trace\left({\hat{C}}_{k}-C_{k}\right)$$

**Figure 10:**
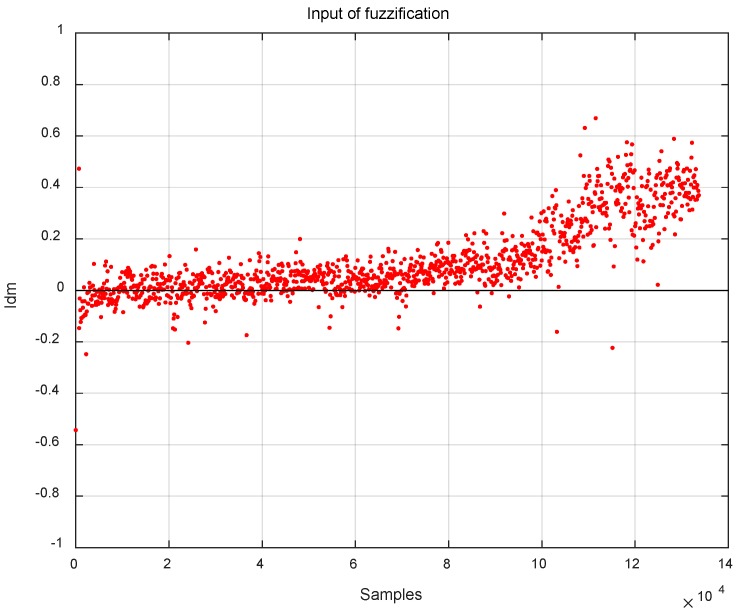
An example of the value of *Idm.*

Figure 10 gives an example that statistical sample covariance of innovation ${\hat{C}}_{k}$ traces its theoretical value *C_k_* and the value of *Idm_k_* is equal to zero at a period of time from the beginning of the sampling when EKF is in an optimal mode. However, the statistical properties of the process noise covariance matrix *Q* will change, causing the value of *Idm_k_* to deviate from zero after a certain time. If no measurements are taken for EKF at this time, systematic state estimation would be biased, and one-step prediction covariance could not truly reflect the change of estimation accuracy, which leads to filter divergence.

**Figure 11:**
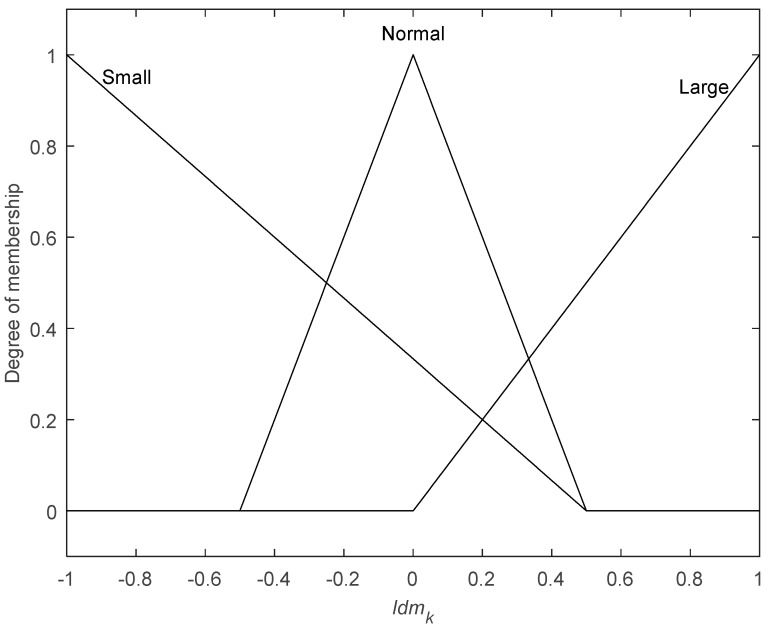
Member function of input *Idm.*

**Figure 12:**
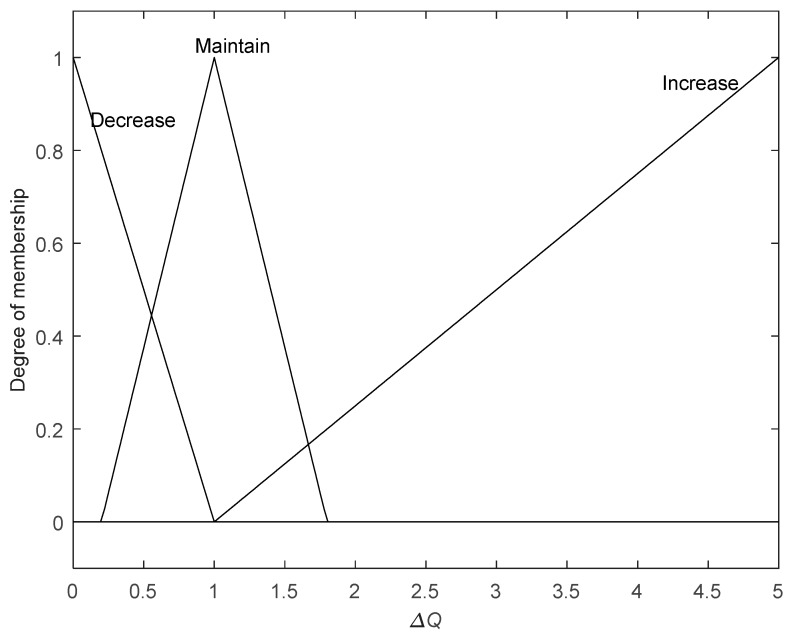
Member function of output Δ*Q.*

The universe of discourse of FIS input *Idm_k_* and output Δ*Q_k_* are [−1,1] and [0,5], respectively. FIS input *Idm_k_* is fuzzified with triangular MFs and mapped to three fuzzy sets {Small, Normal, Large} as Figure 11. FIS output Δ*Q_k_* is also fuzzified with triangular MFs and mapped to three fuzzy sets {Decrease, Maintain, Increase}, as shown in Figure 12. The shape of MFs has a great influence on the performance of FIS. Usually, when the error is small, MFs can be narrow and thin; otherwise, they can be wide and fat.

### 4.2. Fuzzy Inference

Fuzzy inference is the process taking a set of fuzzy rules as the premise, then exerting fuzzy inferential strategy to draw a fuzzy conclusion. The characteristics of FIS depend on the fuzzy rules by which the performance of FAEKF is directly affected. The presented FIS contains three fuzzy rules. If the statistical sample covariance of innovation ${\hat{C}}_{k}$ lies approximately to its theoretical value *C_k_*, it means that the two covariances match well and FIS output Δ*Q_k_* equals one. If the statistical sample covariance of innovation ${\hat{C}}_{k}$ is greater than its theoretical value *C_k_*, FIS output Δ*Q_k_* should be increased. On the contrary, if the statistical sample covariance of innovation ${\hat{C}}_{k}$ is less than *C_k_*, FIS output Δ*Q_k_* should be decreased. Fuzzy rules can also be called fuzzy implication or expressed by the if-then form:

If $\sum_{i=13}^{15}Idmk\left(i,i\right)$ ∈ antecedent, then Δ*Q_k_* ∈ consequent.

With antecedent and consequent denoting fuzzy sets, the following three fuzzy rules (R*i*, *i* = 1,2,3) are used:

R1: If $\sum_{i=13}^{15}Idmk\left(i,i\right)$ ∈ Large, then Δ*Q_k_* ∈ Increase;

R2: If $\sum_{i=13}^{15}Idmk\left(i,i\right)$ ∈ Normal, then Δ*Q_k_* ∈ Maintain;

R3: If $\sum_{i=13}^{15}Idmk\left(i,i\right)$ ∈ Small, then Δ*Q_k_* ∈ Decrease.

After *Idm_k_* is fuzzified and mapped into the fuzzy set ${Idm}_{k}^{*}$, according to the fuzzy rules (R*i*, *i* = 1,2,3), the fuzzy output set Δ$Q_{k}^{*}$ is expressed as below:

(29) $$\Delta Q_{k}^{*}=Idm_{k}^{*}\circ \left(R1\cup R2\cup R3\right)$$

where ∪ is the parallel set in fuzzy mathematics; ∘ represents the max-min composition rule of the fuzzy.

### 4.3. Defuzzification

The output of fuzzy inference in the previous section is the fuzzy set. However, the parameter that needs to be adjusted in the PNS system is deterministic value *Q*. The last step for FIS is defuzzification that transforms the fuzzy output set Δ$Q_{k}^{*}$ into the deterministic value Δ*Q_k_*. Different fuzzification methods yield different results. We choose the centroid to calculate the deterministic value Δ*Q_k_*—the center of the area which is enclosed by MF curve.

Δ*Q_k_* is only served to update the elements in *Q_k_* that correspond to the system noise variance adaptively after FIS completed at each time *k*. Therefore:

(30) $$Q_{k}\left(i,i\right)=Q_{k}\left(i,i\right)\cdot \Delta Q_{k}\;\;\;i=10,11,12$$

**Figure 13:**
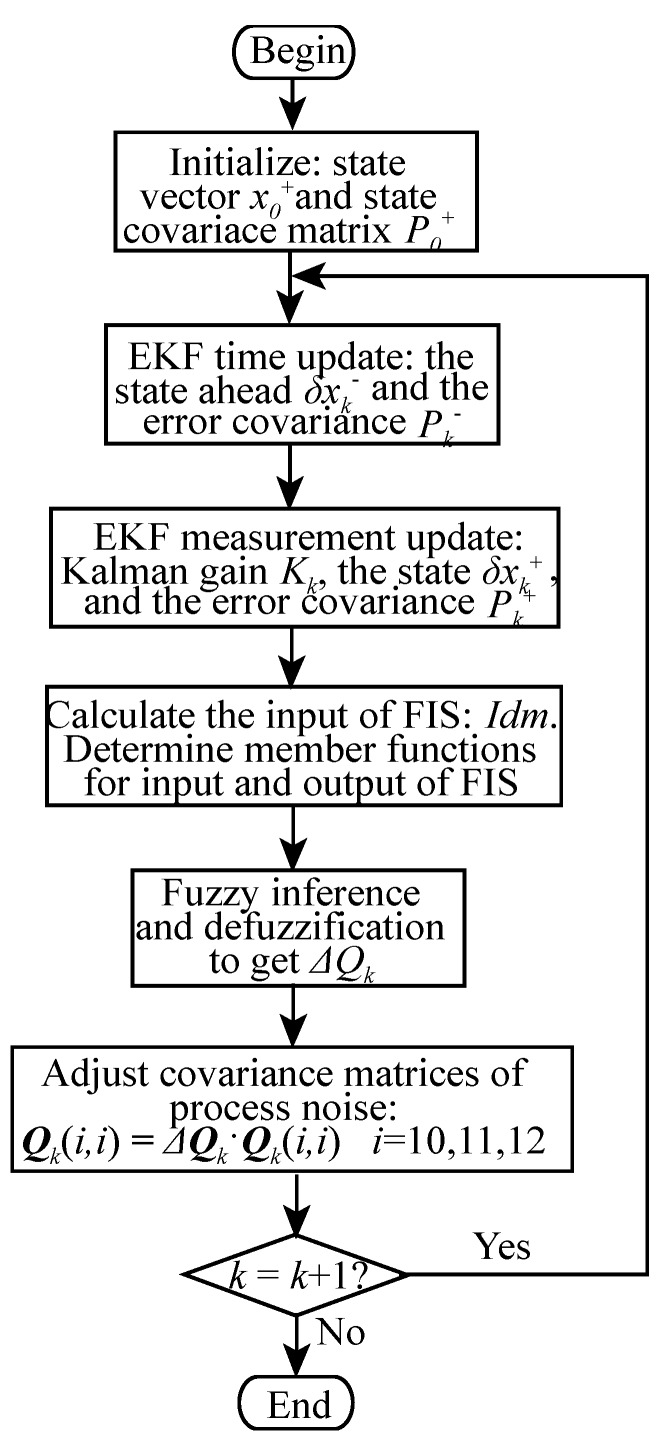
Flowchart of FAEKF.

The flowchart for FAEKF is shown in Figure 13.

## 5. Experimental Analysis, Results, and Discussion

### 5.1. Hardware Description

The MEMS-IMU used in experiments is an MTi-3 (Xens, Enschede, the Netherlands) that consists of a triad of accelerometers, a triad of gyroscopes and a triad of magnetometers with a size of 12.1 × 12.1 × 2.55 mm. More specifications can be seen in Table 1. The ultrasonic sensor is a KS102 (Dauxi Technologies Co., Ltd, Shenzhen, China) with a range of 13–8 m. The MEMS-IMU and the ultrasonic sensor are integrated by an ARM STM32 (AVR, STMicroelectronics, Geneva, Switzerland) mounted on the right shoe. The raw data saved by the processing board are processed offline on the MATLAB platform.

### 5.2. Results and Analyses

Two experiments are carried out to verify the proposed algorithm in different environments. We use errors in percentage of TTD and the absolute error to evaluate the performance of the algorithm. The absolute error is the distance difference between the initial and final positions in a closed-loop trajectory. The error rate of TTD is defined as the percentage error of the absolute error and total travelled distance in a closed-loop trajectory:

(31) $$Error\;rate\;of\;TTD=\frac{\left\|P\left(begin\right)-P\left(end\right)\right\|}{D}\times 100\%$$

where *D* is the total travelled distance; *P*(*begin*) is the position of origin and *P*(*end*) is the position of end; || || is the 2-norm.

**Figure 14:**
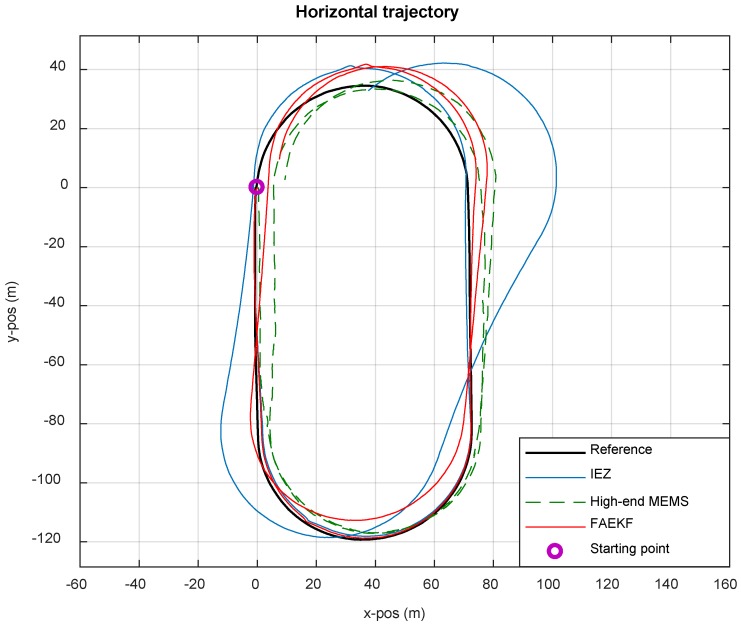
Estimated horizontal trajectories of PNS with different methods.

In the first experiment, the tester walks two laps along the track (800 m) at Beihang University. The results of this experiment are shown in Figure 14. In the navigation coordinate system, the *X* axis and the *Y* axis are in the m point to the East and the North, respectively. The black line is the actual track of the runway as the reference. The tester walks from the starting point (purple circle) in the counterclockwise direction and returns after two rounds.

The blue line represents the moving trail of the pedestrian based on the traditional IEZ. The overall performance of the first cycle is satisfactory. Starting from the second cycle, the performance of the filter tends to deteriorate over time since there is no updating of filter parameters. If IEZ is assisted by FIS, the estimated pedestrian walking path becomes more accurate since the filter parameters are adjusted adaptively. The red line, which takes the proposed algorithm FAEKF, works robustly for FIS based on innovation is used to calculate the estimations of the process noise covariance matrix. The green dashed line, which has high positioning accuracy and robustness, is the estimated horizontal trajectory by means of the high-end MEMS-IMU with stable performance parameters. Experimental results show that FAEKF performs accurately with uncertain noise covariance, and the low-cost MEMS-IMU with FAEKF has the same order of magnitude accuracy as the high-end MEMS-IMU.

Table 2 shows the error of different algorithms. Following the traditional IEZ algorithm, the positioning performance of the low-cost MEMS-IMU with FAEKF is obviously improved and the error rate of TTD decreases to 1.54%. The high-end MEMS-IMU achieves the best performance in that the error in percentage of TTD is 1.04%. Compared to the other two algorithms, the proposed algorithm in this paper has the best comprehensive performance: with the same order of positioning accuracy, the price is lower than that of the high-end MEMS-IMU.

**Figure 15:**
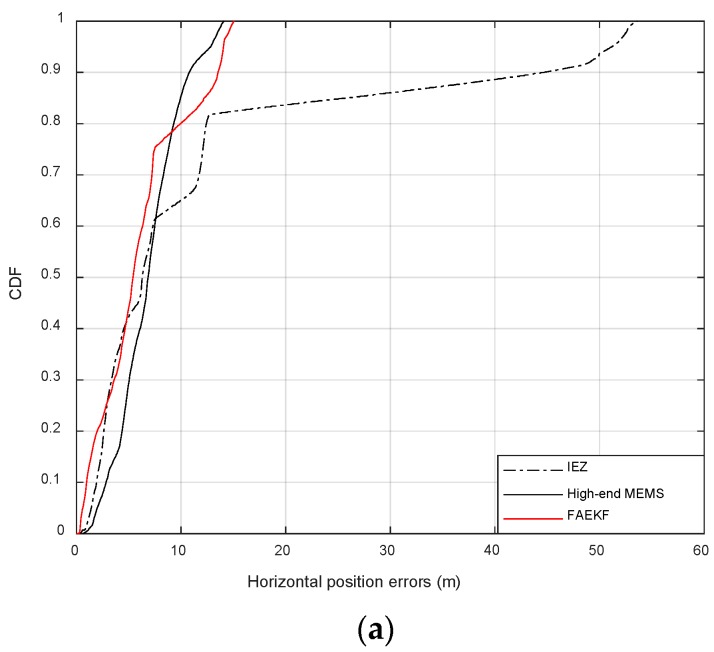

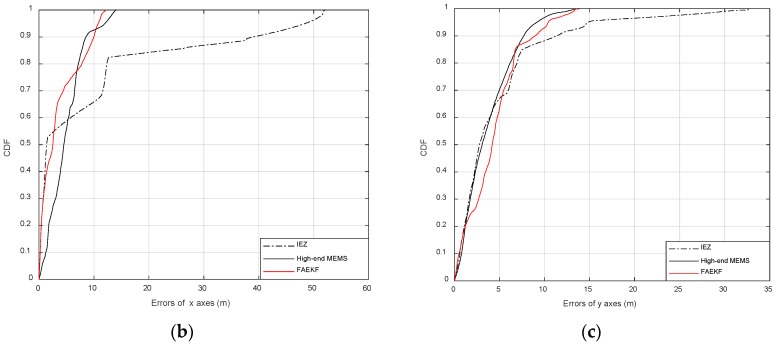

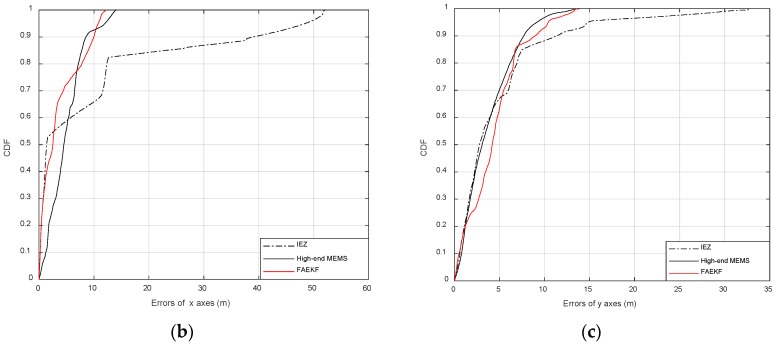
(**a**) CDF about horizontal position errors; (**b**) CDF about errors of *x* axes; (**c**) CDF about errors of *y* axes.

Figure 15 shows the results of the CDF regarding the positioning errors of the first experiment. The red line, the black line and the black dashed line in each subgraph represent the cumulative error distribution curves obtained by the methods of IEZ, high-end MEMS-IMU and FAEKF, respectively.

In Figure 15a, the abscissa represents the horizontal displacement error during the pedestrian positioning. The red line is close to the black line. The error distribution range of the black line is 0–14.10 m, and the one of the red line concentrates on the range of 0–14.93 m. This means that the CDF of errors about the horizontal position with FAEKF is similar to that with the high-end MEMS-IMU. Nevertheless, the black dashed line has a large error distribution (0–53.31 m).

In Figure 15b,c, the CDFs of errors of *x* axes and *y* axes in the process of pedestrian positioning are given. The red line is close to the black line. The error distribution range of the red line is relatively small and concentrated (0–13.52 m in Figure 15b, and 0–13.68 m in Figure 15c), while the black dashed line has a large error distribution (0–51.83 m in Figure 15b, and 0–32.61 m in Figure 15c).

Compared with IEZ, the low-cost MEMS-IMU with FAEKF has achieved higher accuracy which reaches the same level as that of the high-end MEMS-IMU.

**Figure 16:**
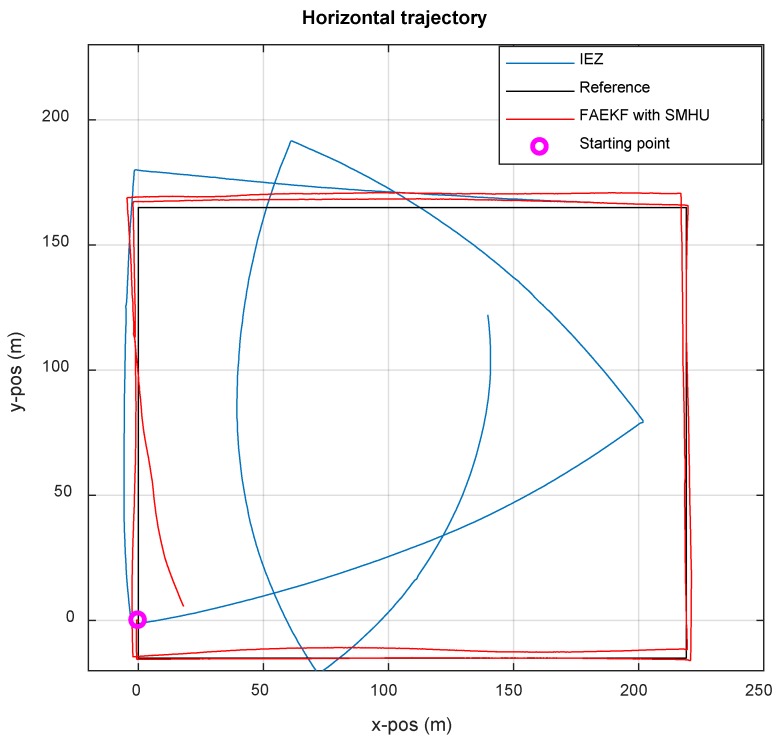
Comparison of position estimates with IEZ and FAEKF methods.

Figure 16 shows the results of the experiment in which the tester wears the hardware on the left foot and walks around the New Main Building of Beihang University for two rings about 1600 m. In the navigation coordinate system, the *X* axis and the *Y* axis in the m point to the East and the North, respectively.

The black rectangle, the planned route in advance and the coordinates measured by the total station, represents the actual reference; the red and blue lines represent the two trajectories estimated by the proposed method and IEZ, respectively. The estimated tracks start from the purple point and change counterclockwise. At the beginning, both the low-cost MEMS-IMU with IEZ and FAEKF with SMHU work normally and filter parameters meet the optimal estimation requirements. After half a cycle, the orientation of the IEZ positioning method begins to shift the actual position, and the positioning performance is not optimal due to the lack of heading measurements and the influence of time-varying process noise covariance matrix Q. For the proposed algorithm, the line information detected by SMHU is used for heading correction and the process noise covariance matrix Q is revised by FIS, so the performance indexes including robustness and accuracy of the estimated location are improved in the case of increased hardware complexity. Compared with IEZ and the proposed algorithm, the errors in percentage of TTD and the absolute errors are 1.14%, 19.0 m and 11.2%, 185.4 m, respectively. The results indicate that FAEKF with SHMU can achieve high performance for PNS in terms of accuracy and robustness.

## 6. Conclusions

In this study, two auxiliary methods are developed to improve the performance of low-cost MEMS-IMU in PNS. The proposed methods feature two strengths: SMHU reduces the heading drift and FIS increases the robustness for PNS.

Heading errors due to the gyro drift of the vertical axis are not observable at each ZUPT state. The proposed ultrasound sensor detects the distance between the wall and pedestrian for recognition of linear trajectory. After conducting experiments, we conclude that position errors are reduced drastically by SMHU. The limitation of SMHU lies in the effective range of ultrasonic sensor and is not suitable for use in halls or other open areas.

The second proposed method consists of FAEKF. Statistical characteristics of process noise change for low-cost MEMS-IMU after long time running. The performance of EKF declines and the filter diverges without boundary. FIS is exploited as an augmentation to the conventional EKF solution in order to adjust the process noise covariance matrix. The major effect of FIS is its simplicity, possibility of exploiting heuristic knowledge, and robustness of the algorithm. Nevertheless, linearization error exists in FAEKF as EKF neglects the higher order term when approximating a nonlinear function.

Furthermore, the proposed algorithm requires no installation on environment. It can also function well in complex environments, such as the field of fire. Future research work related to this study will focus on two ultrasonic sensors which point to different sides (left and right). By means of two ultrasonic sensors, the linear features of the two sides of the corridor will be recognized, especially when one side loses the tracking line due to the absence of walls. In addition, barometer will be fused to expand FAEKF to a three-dimensional positioning system.

## Figures and Tables

**Table 1:** Technical specifications of the angular rate and the acceleration sensors in MTi-3.

Gyroscope		Accelerometer	
Output Data rate	100 Hz	Output Data rate	100 Hz
Bias stability	10^o^/h	Bias stability	0.1 mg
Full-scale range	± 2000^o^/s	Full-scale range	±16 g
Noise density	0.01^o^/s/√Hz	Noise density	200 µg/√Hz
Non-linearity	0.1% of FS	Non-linearity	0.5% of FS

**Table 2:** Comparison of performance with the walking of 800 m.

Algorithm	Evaluation Indicator	
	% of TTD	Absolute Error (m)
IEZ	7.43%	59.02
high-end MEMS-IMU	1.04%	9.33
FAEKF	1.54%	12.20
